# Exome sequencing identifies a missense mutation in *Isl1 *associated with low penetrance otitis media in dearisch mice

**DOI:** 10.1186/gb-2011-12-9-r90

**Published:** 2011-09-21

**Authors:** Jennifer M Hilton, Morag A Lewis, M'hamed Grati, Neil Ingham, Selina Pearson, Roman A Laskowski, David J Adams, Karen P Steel

**Affiliations:** 1 Wellcome Trust Sanger Institute, Hinxton, Cambridge CB10 1SA, UK; 2 Current address: NIH, NIDCD, Bethesda, MD 20892, USA; 3 EMBL European Bioinformatics Institute, Hinxton, Cambridge CB10 1SD, UK

## Abstract

**Background:**

Inflammation of the middle ear (otitis media) is very common and can lead to serious complications if not resolved. Genetic studies suggest an inherited component, but few of the genes that contribute to this condition are known. Mouse mutants have contributed significantly to the identification of genes predisposing to otitis media

**Results:**

The dearisch mouse mutant is an ENU-induced mutant detected by its impaired Preyer reflex (ear flick in response to sound). Auditory brainstem responses revealed raised thresholds from as early as three weeks old. Pedigree analysis suggested a dominant but partially penetrant mode of inheritance. The middle ear of dearisch mutants shows a thickened mucosa and cellular effusion suggesting chronic otitis media with effusion with superimposed acute infection. The inner ear, including the sensory hair cells, appears normal. Due to the low penetrance of the phenotype, normal backcross mapping of the mutation was not possible. Exome sequencing was therefore employed to identify a non-conservative tyrosine to cysteine (Y71C) missense mutation in the *Islet1 *gene, *Isl1^Drsh^*. Isl1 is expressed in the normal middle ear mucosa. The findings suggest the *Isl1^Drsh^*mutation is likely to predispose carriers to otitis media.

**Conclusions:**

Dearisch, *Isl1^Drsh^*, represents the first point mutation in the mouse *Isl1 *gene and suggests a previously unrecognized role for this gene. It is also the first recorded exome sequencing of the C3HeB/FeJ background relevant to many ENU-induced mutants. Most importantly, the power of exome resequencing to identify ENU-induced mutations without a mapped gene locus is illustrated.

## Background

Inflammation of the middle ear mucosa associated with fluid accumulation is known as otitis media [1]. It is very common, being the most frequent cause of surgery in children in the developed world. A recent European cohort reports 35% of children had at least one episode of otitis media before the age of 2 years [2], while a North American cohort found 91% of children did [3], and a range of 50 to 85% of 3 year olds with one or more episodes has also been reported [4]. Otitis media can, however, lead to serious complications, including death [5]. Heritability studies-for example, twin and triplet studies-suggest that otitis media has a significant genetic component [6]. Therefore, studying the causes of otitis media must include exploration of the genetic factors involved.

Otitis media can be caused by Eustachian tube dysfunction due to anatomical blockage or mucocilliary dysfunction [1]. Alternatively, it can be caused by more systemic factors, such as immune dysfunction, healing or complications from a bacterial load that cannot be cleared adequately. Genes affecting any of these processes may cause or predispose to otitis media, meaning that patients affected by variation in one gene may all show otitis media, while variation in another gene may result in only some patients displaying otitis media [7]. Otitis media may be acute (short-lived) or chronic (long lived). Chronic otitis media can also be divided by tympanic membrane pathology into chronic suppurative otitis media (where the tympanic membrane is affected, usually being perforated) or chronic otitis media with effusion (where the tympanic membrane is normal) [8].

Here we report the identification of a new *N*-ethyl-*N*-nitrosourea (ENU)-induced mutation, dearisch, in the mouse by exome sequencing. ENU is a chemical mutagen that, when injected into male mice, mutagenizes spermatogonia, resulting in random point mutations. The dearisch mutant arose from a large scale ENU mutagenesis program looking for new dominant mutations causing hearing loss by screening the first (F1) generation of offspring from ENU-exposed male mice [9]. Previous reports have shown ENU mutants to be a rich source of mouse models of otitis media [10-12]. For example, the Jeff mouse mutant shows fully penetrant chronic proliferative otitis media and a mutation in the *Fbxo11 *gene was identified as being causative. In this case, outcross/backcross mapping followed by sequencing of the locus was used to identify the causal mutation [13]. *Fbxo11 *has since been shown to affect the *TGF-β *pathway [14] and susceptibility to otitis media associated with mutations in this gene have been reported in humans [15]. Another example is the Junbo mutant, which carries a mutation in the *Evi1 *gene. This mutant exhibits acute otitis media leading to chronic suppurative otitis media in most mice [11].

Genetically induced propensity to spontaneous chronic otitis media has been studied in several other mouse mutants, including those with mutations in the genes *Fgfr1 *[16,17], *Trp73 *[18], *Nfkb *[19], *E2f4 *[20], *Eya4 *[21], *Nf2 *[22], *Plg *[23], *Tbx1 *[24], *Rpl38 *[25] and *Scx *[26]. Mutations in the genes *Sall4 *[27], *Sh3pxd2b *[28] and *Phex *[29] have also been implicated in otitis media in mice, but have not been fully characterized. Mutations that lead to immune or autoimmune conditions can also increase susceptibility to otitis media following exposure to bacteria, such as in *Tlr2 *[30], *Tlr4 *[31,32], *Myd88 *[33], *Ticam1 *[34] and *Fas *[35] mutants. Genes that lead to ciliary defects, such as *Gusb *[36], *Idua *[37], *Naglu *[38], *Cby1 *[39] and *Dnahc5 *[40], among others, are known to lead to spontaneous chronic otitis media. As in humans, trisomy 21 can lead to otitis media in mouse mutants, such as Ts65Dn [41]. In humans many candidate genes have also been identified that are suspected of leading to otitis media, including *FBXO11 *[15], *SMAD2, SMAD4, TLR4 *[42], *MUC5AC *[43], *IL6 *[44], *IL10, TNFα *[45], *TGF-β1, PAI1 *[46], *MLB2, G45D *[47], *SP-a1 6A *[48], *CD14 *[49], *IFNγ *[44], *HLA-A2 *[50]*, HLA-A3, G2m(23) *[51] and more.

Identification of mutations causing a phenotype in ENU-induced mouse mutants has traditionally included mapping of backcross progeny to identify the mutated gene. Although this approach has been successfully used to identify many fully penetrant mutations, it requires a reasonable number of affected offspring and is difficult in mutants with low penetrance. Exome sequencing has been successfully used to identify mutations causing genetic conditions in human families despite small pedigrees [52,53]. The use of exome sequencing in mice obviates the need for backcross mapping and is therefore an ideal tool to identify mutations in mutants having complex and/or partially penetrant phenotypes.

The mouse mutant discussed in this paper, dearisch (*Drsh*), was discovered to gradually lose the Preyer reflex (earflick in response to sound), suggesting hearing loss. We report that the low penetrance hearing impairment of dearisch mutants is associated with chronic otitis media and by using exome sequencing we have identified the likely causative mutation in the gene *Islet 1 *(*Isl1*).

## Results and discussion

### Dearisch mice show impaired auditory responses and middle ear inflammation

**Figure 1:**
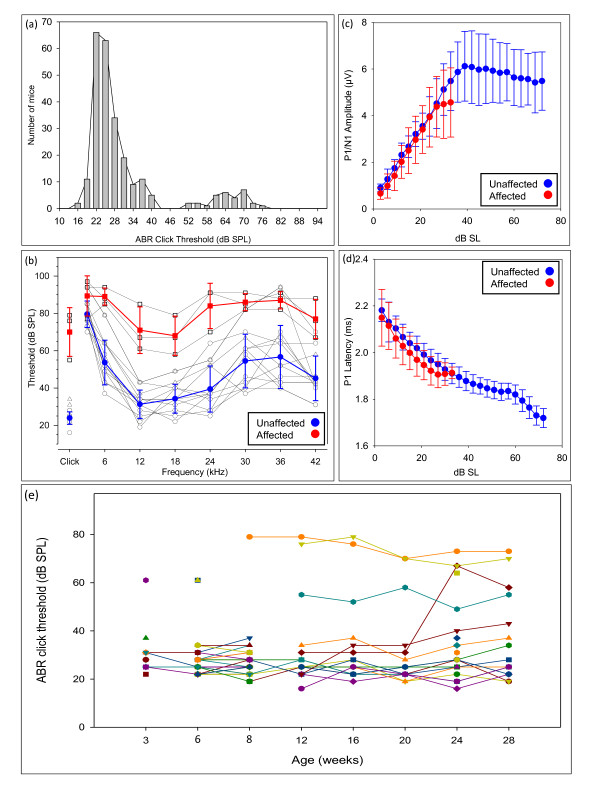
**Auditory brainstem responses in dearisch mice**. **(a) **The distribution of click thresholds of mice in the dearisch colony born between 2009 and 2011 (n = 250). The majority of mice hear normally; however, there is a second peak of mice with a spread of thresholds between 50 and 80 dB SPL. **(b) **The audiograms of mice examined with the long ABR protocol at 12 weeks of age (n = 16). The mean thresholds at each frequency and standard deviation at each frequency for the mice with an ABR click threshold above 50 dB SPL (affected) and below 30 dB SPL (unaffected) are shown in red and blue, respectively. The shape of the mean affected audiogram is similar to the unaffected audiogram with approximately 40 dB increase in threshold (hearing loss) at each frequency, consistent with a conductive hearing impairment. **(c) **Growth of ABR wave 1 amplitude with increasing stimulus intensity, plotted as dB above threshold (sensation level, dB SL), is similar in affected and unaffected mice, consistent with a purely conductive defect; n = 13 affected mice (in red) and 13 unaffected mice (in blue). **(d) **Reduction in latency to the first peak of the ABR waveform with increasing stimulus intensity above threshold (dB SL) is similar in affected and unaffected mice, consistent with a conductive defect; n = 13 affected mice (in red) and 13 unaffected mice (in blue).**(e) **Measurement of click-evoked ABR thresholds with recovery allowing repeated ABR measurements in individual mice with increasing age from 3 to 28 weeks. From 8 to 28 weeks 16 mice underwent recurrent recordings and 9 mice underwent single recordings. Between 3 and 8 weeks a different set of mice (n = 66) underwent one or two click ABR recordings. Although there is some variability in thresholds, most mice could hear normally, while a few mice have raised thresholds from as early as 3 weeks. In general, thresholds are stable, not increasing with age.

We distinguished affected mice in the dearisch colony by auditory brainstem response (ABR) threshold measurements. Mice display a range of ABR thresholds to click stimuli, from normal (approximately 15 to 30 dB sound pressure level (SPL)) to moderate hearing impairment (between 50 and 80 dB SPL), with a bimodal distribution (n = 250; Figure 1a). Affected mice were defined as having a click threshold of 50 dB SPL or over, and mice with click thresholds of 30 dB SPL or below were defined as unaffected mice. Measurements of thresholds at a range of frequencies at 12 weeks old showed approximately 40 dB hearing loss across the majority of frequencies in affected mice (Figure 1b). This consistent loss across frequencies, mirroring the shape of the audiogram in unaffected, hearing mice, associated with a hearing loss of rarely more than 40 dB and normal growth of waveform amplitudes and reduction in latencies with increasing stimulus intensity above threshold (Figure 1c, d), are all consistent with conductive pathology as the most likely cause for the hearing impairment.

Repeated ABR testing on a cohort of aging mice demonstrated that affected dearisch mice have hearing impairment from the earliest age tested (3 weeks), and this surprisingly does not generally progress with age (Figure 1e).

**Figure 2:**
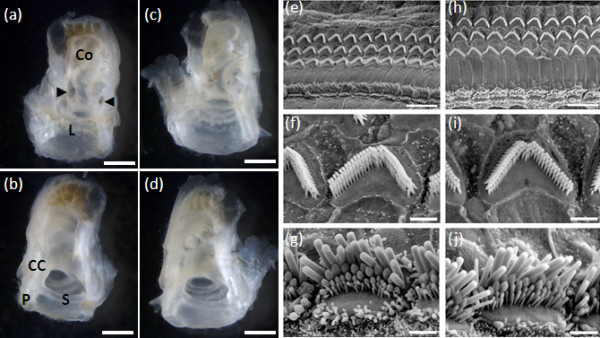
**Inner ear in dearisch mice (a-d) Inner ears show no sign of abnormal gross morphology: (a, b) unaffected mouse; (c, d) affected dearisch mouse**. (a, c) Inner ear viewed from the middle ear side. (b, d) Inner ear viewed from the brain side. The leftwards-pointing arrowhead indicates the round window and the rightwards-pointing arrowhead indicates the oval window; CC, common crus; Co, cochlea; L, lateral semicircular canal; P, posterior semicircular canal; S, superior semicircular canal. **(e-j) **Scanning electron microscopy at 50% of the distance along the length of the organ of Corti showing normal ultrastructure: (e-g) from unaffected mouse; (h-j) from affected dearisch mouse. (e, h) Normal organ of Corti layout with three rows of outer hair cells and one row of inner hair cells. (f, i) Outer hair cells with a normal morphology. (g, j) Normal inner hair cells. The whole length of the organ of Corti was examined at 10% intervals and no abnormalities were detected (data not shown). Scale bars: 1 mm (a-d); 10 μM (e, h); 1.5 μm (f, g, I, j).

Gross anatomy of the inner ear appears normal (Figure 2a-d) and the round and oval window areas are not significantly different between unaffected and affected mice (Student's *t*-test; *P*-value 0.24 and 0.86, respectively; data not shown). Ultrastructural anatomy of the cochlea assessed using scanning electron microscopy shows normal sensory hair cell morphology and layout (Figure 2e-j).

**Figure 3:**
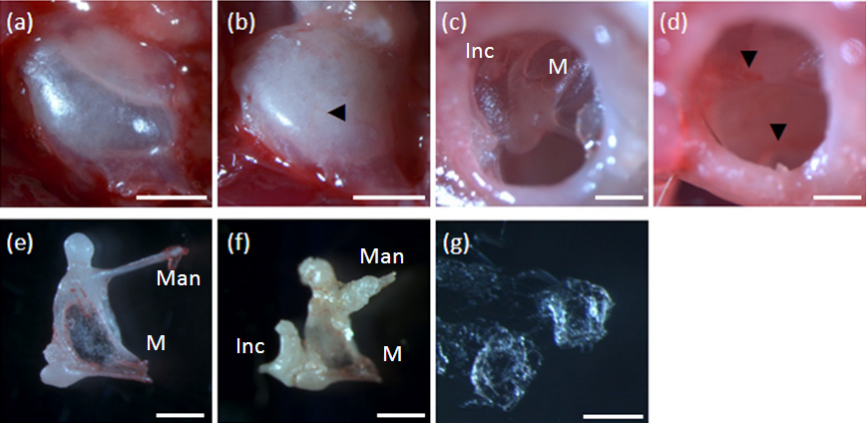
**Histology of the middle ear**. **(a) **A normal unaffected translucent bulla in an unaffected animal. **(b) **An abnormally white bulla with a small engorged capillary (indicated by the arrowhead) from an affected animal. **(c) **An unaffected animal with a normal transparent tympanic membrane and the malleus (M) and incus (Inc) visible beneath. **(d) **The tympanic membrane is opaque with engorged capillaries on the surface (indicated by arrowheads). This animal also showed raised ABR thresholds. **(e) **A normal malleus from an unaffected animal. **(f) **A malleus (M) with fused incus (Inc) and extraneous bony growth on the malleus head and manubrium (Man) from an affected animal. This represents the most extreme example of extraneous bony growth. **(g) **Crystalline deposits found in the middle ear cavity of an affected animal. Scale bars: 1 mm (a, b); 0.5 mm (c-f); 0.2 mm (g).

However, middle ear examination revealed chronic otitis media with an intact tympanic membrane (Figure 3). Affected mice displayed a variety of pathological features associated with otitis media, including: white bony bulla instead of translucent bone (12 of 14); an abnormally vascularized bulla (5 of 14); a vascularized tympanic membrane (5 of 14); fluid in the middle ear-mostly thick, white, opaque, but not sticky fluid (11 of 14); mucosal oedema (6 of 14); crystalline deposits around the malleus (6 of 14); bony outgrowths that sometimes included fusion of ossicles (9 of 14); and excessive cerumen in the external ear canal (12 of 14). The severity of otitis media was variable and this may account for the variability of the ABR findings. The ABR thresholds did not fluctuate substantially in most individual mice over time (Figure 1c), implying the hearing impairment is due to chronic middle ear disease rather than recurrent acute otitis media. Middle ears of unaffected mice with normal click thresholds were not entirely normal, and showed some abnormal signs, including: a white bony bulla (2 of 14); a vascularized bulla (1 of 14); a vascularized tympanic membrane with engorged capillaries (1 of 14); fluid in the middle ear, either clear or turbid (4 of 14); edema of the middle ear lining (1 of 14); crystalline deposits (4 of 14); bony overgrowths (2 of 14); and cerumen in the external auditory canal (5 of 14). Mild and less frequent pathology in mice with normal thresholds is not entirely unexpected, as the apparent reduced penetrance of the phenotype means some hearing mice will carry the mutated gene and may exhibit some features of otitis media without this being severe enough to compromise ABR thresholds.

**Figure 4:**
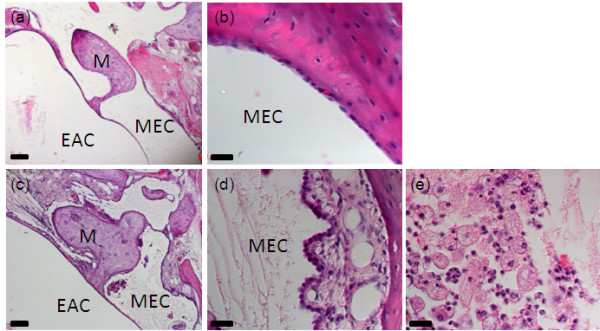
**Hematoxylin and eosin staining of the middle ear in adult mice**. **(a, b) **The middle ear of an unaffected animal. This has a clear middle ear cavity (MEC), external auditory canal (EAC) and a thin, single cell mucosal lining of the cavity. **(c, d) **An affected animal with a normal EAC, but effusion within the MEC and a thickened mucosa, with fibroblasts, granulocytes and granulation tissue. **(e) **A magnified view of the effusion in an affected animal, containing foamy macrophages and neutrophils. M, malleus. Scale bars: 100 μm (a, c); 20 μm (b, d, e).

Histology of normally hearing mice revealed a single cell thick mucosa lining the middle ear, while in affected mice there was evidence of thickened mucosa with fibrocytes, granulocytes and granulation tissue (Figure 4). This is typical of chronic otitis media. The middle ear cavity of affected mice contained cellular effusion including foamy macrophages and neutrophils, suggesting an acute, possibly infective, otitis media superimposed upon the chronic otitis media. While no unaffected mice grew any bacteria on culture of external and middle ear swabs, two out of four affected mouse middle ears and one out of four of their external ear canals grew *Proteus *sp. (DJ Pickard, personal communication)

### Autosomal dominant inheritance with reduced penetrance of hearing impairment

The current dearisch colony is derived from a single male on a C3HeB/FeJ background. This original founder male had mild hearing loss (click threshold 34 dB SPL) on ABR, suggesting variable expressivity of the mutation. When crossed with known wild-type females from the original C3HeB/FeJ background, the male produced some mildly and some moderately affected offspring in the F1 generation, suggesting dominant inheritance. The male was able to produce both affected male and female progeny, suggesting that X-linked inheritance is unlikely. The colony has been outcrossed at least five times to wild-type mice from a C3HeB/FeJ colony that had not been exposed to ENU, diluting out ENU-induced mutations that are unrelated to the dearisch phenotype. There were smaller numbers of affected mice in the colony than could be explained by a simple Mendelian model with full penetrance.

We attempted to map the mutation by outcrossing an affected male to C57BL/6J females, then backcrossing affected outcross offspring to known wild-type C57BL/6J mice. Five affected outcross mice were found out of 168 tested, but when these were backcrossed there were no affected backcross offspring out of 77 tested so we were unable to map the mutation by the usual linkage analysis approach.

### Exome resequencing identifies an *Isl1 *missense mutation

We used the Agilent SureSelect *^XT ^*mouse all exon kit for sequence capture followed by Illumina Genome Analyzer II next-generation sequencing to search for the causative mutation using one DNA sample from an affected dearisch mouse and one sample from the C3HeB/FeJ colony (Table 1). Agilent reports 49.6 Mb capture of 221,784 exons from 24,306 genes using this kit [54]. Sequencing reads were mapped to NCBI build 37 of the mouse genome (C57BL/6J) using bwa 0.5.7 [55] and duplicate fragments were marked using picard 1.15 [56]. SAMtools 0.1.8 [57] was used to obtain a list of single nucleotide variants (SNVs) and short insertions and deletions. These were filtered to remove variants found in both wild-type (C3HeB/FeJ) and dearisch mutant sequences, and then to remove variants known to be present in other strains, from dbSNP (build 128 [58]) [59] and from the resequencing of 17 inbred strains [60] (Table 2). Variants were finally filtered on the basis of SNP quality (with a lower limit of 20), mapping quality (with a lower limit of 45) and read depth (with a lower limit of 10). This resulted in approximately 8,000 variants. These were then prioritized on the basis of type and consequence. Those SNVs that were predicted to cause either the gain or loss of a stop codon, that resulted in an amino acid change in the protein or that were within an essential splice site (defined as being in the first or last two base pairs of an intron) were chosen for further analysis. There were 23 SNVs that fitted these criteria (Tables 2 and 3).

**Table 1:** Details of exome sequencing results

Sequencing details	C3HeB/FeJ	Dearisch
Type of sequencing	Paired end	Paired end
Read length	76 bp	76 bp
Number of reads mapped	96603761	96517342
Mean depth	126.24×	125.97×
Coverage of bases in Agilent exons	99.71%	99.68%
Coverage of bases in Agilent exons to a depth of 10 fold or more	98.28%	98.05%
Coverage of bases in Agilent exons to a depth of 20 fold or more	95.63%	95.17%

**Table 2:** Filtering of exome sequence data to identify the mutation in *Isl1*

Processing steps	Number of DNA changes
Different from the C57BL/6J reference sequence (C3H/*Drsh*)	7261538/7242100
C3H not same as *Drsh*	5022723
Not in dBSNP and 17 wildtype strains	3654870
Samtools quality filter	76264
Mapping quality > 45 and read depth > 10	7980
Remove intronic and intergenic variants	1260
Select stop, nonsynonymous and splice site SNVs	23
Confirm with capillary sequencing	1

**Table 3:** Details of the 23 SNVs analyzed further after filtering of exome sequence data

								Dearisch				C3HeB/FeJ						
							
Gene name	Location	Predicted DNA change	Reference (C57BL/6J)	Consensus	Genotype	Consensus quality	SNP quality	Mapping quality	Read depth	Consensus	Genotype	Consensus quality	SNP quality	Mapping quality	Read depth	Cap seq C3H	Cap seq *Drsh*	Comments
1700001K19Rik	12:111907080	Nonsynonymous: H:L	T	A	Hom	20	51	58	63	T/A	Het	72	134	55	73	Deletion	Deletion	Misalignment around deletion.
1700104B16Rik	8:34841236	Nonsynonymous: H:D	G	G/C	Het	76	76	55	54	G	Hom	9	0	52	58	G/C	G/C	The dearisch read is correct; the incorrect C3H read has a very low consensus score
*Acsl3*	1:78692680	Stop gained	C	A	Hom	7	25	49	16	A/C	Het	9	9	56	15	C	C	Deep sequencing miscalled an A in *Drsh *and a C/A het in C3H. Neither of them have high consensus or SNP quality scores
*Bcl2l14*	6:134377474	Nonsynonymous: N:K	T	G	Hom	3	36	50	64	G/A	Het	9	10	60	61	NA	Deletion	Misalignment around deletion; low quality consensus and SNP scores
*Btnl7*	17:34670007	Nonsynonymous: G:R	C	C/T	Het	6	96	48	30	T	Hom	96	141	53	44	C/T	C/T	The C3H read has been miscalled as a homozygote
*Catsper2*	2:121223476	Nonsynonymous: N:D	T	C	Hom	33	33	47	83	T/C	Het	15	28	44	86	Deletion	Deletion	Misalignment around deletion
*Col6a3*	1:92672331	Essential splice site	C	G	Hom	30	30	60	16	A	Hom	33	33	29	18	NA	Deletion	Misalignment around deletion
*Creb3l2*	6:37284584	Essential splice site	T	C/T	Het	38	38	54	23	T	Hom	11	0	56	18	T	T	The dearisch read has been miscalled as a heterozygote
*Gm10859*	2:5833494	Nonsynonymous: I:V	A	A/G	Het	41	48	56	18	A	Hom	39	0	41	17	Deletion	Deletion	Misalignment around deletion
*Gm11149*	9:49380322	Nonsynonymous: Q:P	A	C	Hom	0	36	54	30	G/C	Het	0	23	52	30	Deletion	Deletion	Misalignment around deletion and low quality consensus scores
*Gtf3c2*	5:31476808	Nonsynonymous: E:G	T	C/T	Het	25	25	49	39	T	Hom	33	0	52	30	T	T	The dearisch read has been miscalled as a heterozygote. Its consensus and SNP quality scores are low
H2-Oa	17:34229420	Nonsynonymous: V:A	T	C/T	Het	3	35	48	86	T	Hom	39	0	46	79	Deletion	Deletion	Misalignment around deletion
*Ido1*	8:25703857	Nonsynonymous: R:K	C	C/T	Het	21	21	50	30	C	Hom	13	0	53	39	C	C	The dearisch read has been miscalled as a heterozygote. Its consensus and SNP quality scores are low
*Isl1*	13:117098488	Nonsynonymous: Y:C	T	C/T	Het	199	228	60	66	T	Hom	223	0	60	65	T	C/T	Confirmed by capillary sequencing
*Mdc1*	17:35984844	Nonsynonymous: E:D	G	T	Hom	13	39	50	11	G/T	Het	21	21	55	11	G	G	Deep sequencing miscalled a G as a T in *Drsh *and a G/T het in C3H. Neither of them has a very high consensus quality score
*Olfr424*	1:176066876	Essential splice site	A	G/T	Het	4	58	58	88	T	Hom	6	60	60	88	Insertion	A	Misalignment around insertion, also low consensus quality scores
*Olfr573-ps1*	7:110091057	Nonsynonymous: H:Q	G	T	Hom	21	25	56	82	G/T	Het	33	34	56	96	Deletion	Deletion	Misalignment around deletion
*Olfr573-ps1*	7:110091058	Nonsynonymous: H:L	T	A	Hom	22	45	53	79	T/A	Het	8	62	57	96	Deletion	Deletion	Misalignment around deletion
*Olfr749*	14:51356853	Nonsynonymous: Q:K	G	G/T	Het	36	36	46	81	G	Hom	17	0	39	62	Deletion	Deletion	Misalignment around deletion
*Rsf1*	7:104809403	Nonsynonymous: E:Q	G	G/C	Het	17	22	54	47	G	Hom	42	0	55	45	Deletion	Deletion	Misalignment around deletion
*Rsf1*	7:104809404	Nonsynonymous: E:V	A	A/T	Het	14	22	54	47	A	Hom	42	0	55	45	Deletion	Deletion	Misalignment around deletion
*Sap30 bp*	11:115825338	Nonsynonymous: A:T	G	A/G	Het	31	31	55	61	G	Hom	40	0	55	52	G	G	The dearisch read has been miscalled as a heterozygote
U1	1:172958261	Essential splice site	T	A/T	Het	18	105	51	69	A	Hom	26	75	51	58	Deletion	Deletion	Misalignment around deletion

Capillary sequence results for the C3HeB/FeJ and dearisch DNA samples and comments on the reason for each false call are shown in the rightmost three columns. Fourteen of the calls were due to insertions or deletions present at that location that were identical in the two DNA samples, and the original call was due to different nucleotides affected by the deletion being called in the two samples. Het, heterozygous; Hom, homozygous; NA, sequence not available. Only one SNV was confirmed to be present in dearisch and not in C3HeB/FeJ or the C57BL/6J reference sequence, that in *Isl1*.

**Figure 5:**
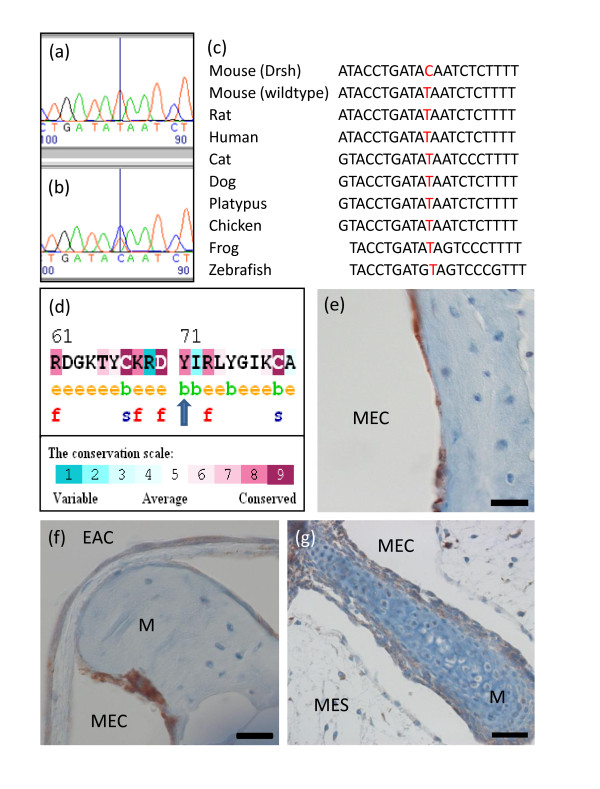
**Islet1 sequence analysis and expression in dearisch mice**. **(a, b) **In the wild-type original background mouse, capillary sequencing confirmed a T/T residue (a), while in affected animals C/T was found (b). No homozygote mutants were identified, suggesting homozygote lethality. **(c) **The thymine base indicated in red was conserved among the species shown and also in giant panda, guinea pig, cow, sloth, armadillo, hedgehog, horse, gorilla, African elephant, mouse lemur, opossum, rabbit, chimp, hyrax, brown bat, common shrew, wild boar, puffer fish, bush baby, dolphin and alpaca (sequences obtained from Ensembl [88]). **(d) **Using ConSurf [89] the tyrosine amino acid residue (indicated by a blue arrow) was found to have a high conservation score of 8, and was predicted to be buried (green letter 'b') rather than exposed (orange letter 'e'). It is not noted as being either structural (blue letter 's') or functional (red letter 'f'); however, it is next to a highly conserved, exposed, functional residue and therefore may be important in positioning this residue. **(e) **Immunohistochemistry using Isl1 antibody indicates expression (brown) within the mucosal lining of the middle ear cavity (MEC) in wild-type adult mice. **(f) **Immunohistochemistry showing Isl1 labeling in the cell layer covering the malleus (M) and the outer layer of the tympanic membrane, adjacent to the external auditory canal (EAC) in the wild-type adult. **(g) **Immunohistochemistry showing more diffuse Isl1 labeling in the cell layer over the malleus at postnatal day 4. The middle ear is still largely filled with mesenchyme (MES) at this early stage. Scale bar: 20 μm (e, f); 40 μm (g).

Of the 23 variants of interest, all were autosomal and 14 were present as heterozygotes, consistent with the expected autosomal dominant pattern of inheritance. All 23 variants were analyzed further by capillary sequencing using the original two DNA samples, which resulted in exclusion of most of the variants as false positive variant calls on the basis that the DNA sample from the mutant DNA was identical to that of the wild-type C3HeB/FeJ DNA at that position (Table 3). The high number of false positives is due partly to the presence of small inserts or deletions causing the SAMtools SNP caller to misread SNVs either side of the indel. Most of the other false positives can be seen to have low consensus and/or SNP quality scores for either or both dearisch and C3HeB/FeJ sequences; SNVs were not filtered on consensus score at all, and only lightly on SNP quality score, because we preferred false positives to false negatives. Only one SNV has high consensus quality, SNP quality, mapping quality and read depth scores, and this has been found by capillary sequencing to be a correct call. This SNV is a point mutation in *Isl1 *leading to a T to C base pair transition at position MMU13:117098488 causing a substitution of tyrosine by cysteine (Y71C; Figure 5a, b). This missense mutation affects an amino acid within the first LIM domain of Isl1.

**Figure 6:**
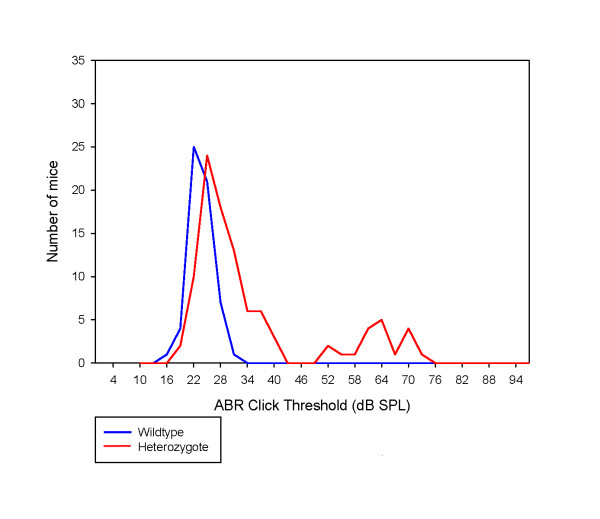
**Distribution of ABR click thresholds in the dearisch colony divided by genotype for the *Isl1^Drsh^*mutation, showing overlapping of heterozygotes (red) and wild types (blue) at low thresholds and heterozygotes only with high thresholds**. Please note on the figure itself in the pdf sent previously (not included in this file) that the legend in the box at the bottom left has lost its red line indicating the heterozygote line.

Capillary sequencing of this position in 21 wild-type strains and in 5 individual C3HeB/FeJ wild-type mice reveals that all are homozygous (T/T) for the reference allele. Indeed, this T to C transition in dearisch mutants alters a tyrosine residue that is highly conserved in orthologous proteins in other species (Figure 5c, d). Having detected this promising candidate mutation, we sequenced DNA samples from throughout the dearisch colony. All 28 affected dearisch mice (born between 2009 and 2011) were heterozygotes (T/C). All of the mice with thresholds above 50 dB SPL were found to have one copy of the *Isl1 *mutation (Table 4). Of the offspring of known heterozygote by heterozygote matings, no pups out of 111 were detected as homozygous for the *Isl1 *mutation, suggesting severely reduced homozygote viability. The penetrance of raised ABR thresholds (> 50 dB SPL) in known heterozygotes is 23.1%. Interestingly, most of the mice with ABR click thresholds of 30 to 50 dB SPL were also heterozygous for the dearisch *Isl1 *mutation (Table 4; Figure 6), giving a penetrance of 51.2% if the more mildly affected mice are included. Furthermore, most of the 'unaffected' mice with thresholds of 30 dB SPL or less but with signs of subclinical middle ear inflammation mentioned earlier were found to be carriers of the *Isl1^Drsh^*mutation (data not shown).

**Table 4:** Analysis of offspring from dearisch matings

	< 30	30 to 50	> 50	Total	Percent
**Het × Het^a^**					
WT	37 (23/15)	2 (1/1)	0 (0/0)	39	35.10%
Het	32 (25/7)	21 (13/8)	19 (13/6)	72	64.90%
Hom	0 (0/0)	0 (0/0)	0 (0/0)	0	0.00%
Total	69	23	19	111	
Percent	62.20%	20.70%	17.10%		
**Het × WT^b^**					
WT	35 (19/16)	1 (1/0)	0 (0/0)	36	42.40%
Het	27 (15/12)	13 (8/5)	9 (6/3)	49	57.60%
Hom	0 (0/0)	0 (0/0)	0 (0/0)	0	0.00%
Total	62	14	9	85	
Percent	72.90%	16.50%	10.60%		

^a^The genotypes and ABR click thresholds of mice from the dearisch colony born to known heterozygote (Het) by heterozygote matings. ^b^The genotypes and click-evoked ABR thresholds of mice from the dearisch colony born to known heterozygote by wild type (WT) matings. The number of mice in each category is given, with number of males/number of females in parentheses. Hom, homozygote.

The close linkage of the *Isl1 *variant with the otitis media phenotype is strong support for this being the causative mutation. However, it remains a possibility that the *Isl1 *variant is simply a linked marker. In order to exclude linkage between the *Isl1 *mutation and any other potentially causative mutation, it is important to exclude other mutations on chromosome 13 (Table 5). Of the 23 SNVs (non-synonymous, stop gained and splice site mutations) identified by exome sequencing, the *Isl1 *mutation is the only one on chromosome 13 (Table 3). Four other chromosome 13 SNVs were excluded at the final filtering step, one in a noncoding transcript of *Tpmt*, one in the 5' UTR of *Smad5 *and two in the 3' UTRs of the genes *Histh1a *and *Sdha*, the closest of which is 70 Mb from the *Isl1 *mutation. We also examined indels from chromosome 13. The SAMtools variant caller identifies short indels as well as SNVs, and these indels were not included in the final analysis of 23 variants. Thirteen deletions and twelve insertions were identified on chromosome 13, although only one and five, respectively, were within coding regions. Of the insertions and deletions within 10 Mb of *Isl1*, none were within coding regions.

**Table 5:** Exclusion of potential linkage within 10 Mb of *Isl1 *(117098488) and on the remainder of chromosome 13

Gene name	Position	Reference	Dearisch	Type	Location	Zygosity	Consequence (if in coding region)^a^
**Within 10 Mb of *Isl1***							
*Ipo11*	107700899	*	-T/*	Deletion	Splice site (intronic)	Het	
*Kif2a*	107752127	*	*/-G	Deletion	3' UTR	Het	
*Slc38a9*	113523874	*	-TT/-TT	Deletion	5' UTR	Hom	
*Gzmk*	113963370	*	*/+T	Insertion	Splice site	Het	
**On remainder of chromosome 13**							
*Pfkp*	6604227	*	*/+GG	Insertion	Exonic	Het	Frameshift leading to truncation of protein in exon 10 (approximately half its length)
*Gtpbp4*	8984980	*	*/-A	Deletion	Splice site (intronic)	Het	
*Pgbd1*	21515496	*	-AGGAA/-AGGAA	Deletion	Splice site (intronic)	Hom	
*Isca1*	21587150	*	*/-GGCTGCGG	Deletion	5' UTR	Het	
*Hist1h1c*	23831772	*	+TN/+TN	Insertion	3' UTR	Hom	
*Hist1h1a*	23856249	A	A/C	SNP	3' UTR	Het	
*Agtr1a*	30473986	*	*/-T	Deletion	3' UTR	Het	
*Txndc5*	38599758	*	*/-A	Deletion	Splice site (intronic)	Het	
*Gm9979*	40801514	*	+CACACACACACG/*	Insertion	3' UTR	Het	
*Tpmt*	47135375	A	A/T	SNP	Noncoding (retained intron)	Het	
*Iars*	49829191	*	*/-TG	Deletion	3' UTR	Het	
*Sema4d*	51798481	*	*/+G	Insertion	Exonic	Het	Frameshift leading to truncation of protein in last exon
*Cdhr2*	54827830	*	*/+G	Insertion	Exonic	Het	Frameshift leading to truncation of protein in exon 19 (approximately two-thirds of its length)
*Smad5*	56824847	*	*/+CACACACACACA	Insertion	5' UTR	Het	
*Smad5*	56824796	C	C/T	SNP	5' UTR	Het	
*Klhl3*	58165232	*	-GA/-GA	Deletion	Splice site (intronic)	Hom	
*Ptch1*	63613020	*	*/+A	Insertion	Exonic	Het	Frameshift leading to truncation of protein very close to carboxyl terminus
*1110018J18Rik*	64393367	*	+A/*	Insertion	3' UTR	Het	
*Sdha*	74460494	T	G/T	SNP	3' UTR	Het	
*Spata9*	76115351	*	-C/-C	Deletion	Exonic	Hom	Frameshift leading to incorrect final 15 amino acids and loss of stop codon
*Fam81b*	76408769	*	+TTA/+TTA	Insertion	Exonic	Hom	Gain of stop codon leading to truncation of protein after 15 amino acids
*Rasa1*	85370111	*	+G/+G	Insertion	Noncoding (retained intron)	Hom	
*Rasgrf2*	92024132	*	-A/-A	Deletion	Splice site (intronic)	Hom	
*Pde8b*	95822955	*	*/-TAA	Deletion	Noncoding (retained intron)	Het	
*Bdp1*	100808235	*	*/+A	Insertion	Splice site (intronic)	Het	

^a^Predicted consequences are shown where the mutation lies in a coding region. Plus signs indicate insertion; minus signs indicate deletion. *Asterisks indicate the presence of the wildtype reference sequence where an insertion or deletion has been called in the dearisch sequence. These calls have not been confirmed by capillary sequencing.

### Isl1 is expressed in the middle ear

We next asked if Isl1 protein is expressed in the middle ear. Immunohistochemistry of the adult wild-type middle ear revealed clear, widespread expression of Isl1 within the single cell mucosal lining of the middle ear cavity, including the single cell layer covering the ossicles, but less pronounced on the inner surface of the tympanic membrane (Figure 5e, f). Expression is also seen in the epithelial layer of the external ear canal and outer layer of the tympanic membrane. At postnatal day 4, the expression is more diffuse but is present in the immature mucosa where the middle ear has cavitated and in the outer cellular layer surrounding the ossicles (Figure 5g).

### Modeling the consequences of the Y71C missense mutation on protein structure

According to Pfam [61], the Isl1 protein consists of four Pfam domains: two LIM domains, a homeodomain and a Gln-rich domain. Each LIM domain contains two zinc fingers, which each bind a zinc atom. The LIM-homeodomain (LIM-HD) combination is thought to represent a 'LIM code' that governs transcriptional regulation in the control of cell type specification in different tissues and organs [62]. Isl1 is a member of the LIM-HD family of proteins. The two LIM domains are responsible for interaction with other proteins while the homeodomain uses its helix-turn-helix motif to bind DNA sequences containing the sequence 5'-ATTA-3' and so initiate transcription of the appropriate genes.

Proteins binding to LIM-HD proteins do so via a LIM-interaction domain (LID), which consists of around 30 residues. The Y71C mutation is located within the first LIM domain and so may affect the strength of this binding. To predict how it might do so requires knowledge of the protein's three-dimensional structure.

To date, there have been no experimental determinations of the three-dimensional structure of Isl1 protein (other than fragments of the carboxy-terminal domain). However, there are many structural models of related proteins in the Protein Data Bank (PDB) [63]. One of these, PDB entry 2xjy, is of particular interest. This is a structural model, solved by X-ray crystallography to 2.4 Å resolution, of human rhombotin-2 (aka LMO2). The protein is a LIM-only (LMO) protein; that is, it consists of two LIM domains only. However, the structure is a complex between this protein and a 35-residue fragment of a LID from human LIM domain-binding protein 1. As such, it provides a general idea of how LIM domains recognize their interaction partner. The three-dimensional structure reveals that the LID fragment binds in an extended conformation along a groove running along the length of the two LIM domains.

**Figure 7:**
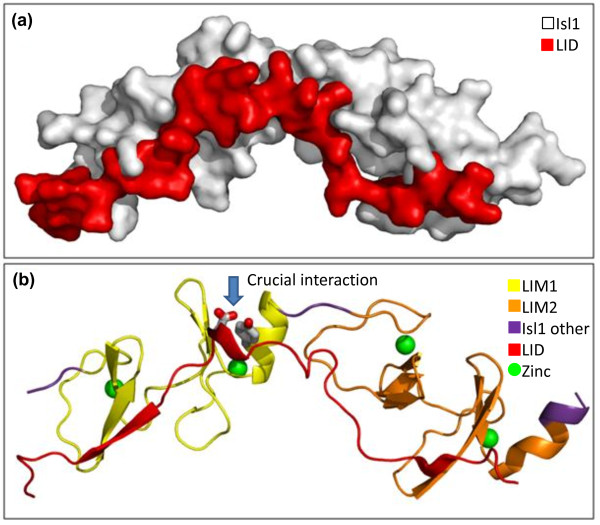
**A homology model of ISL1 based on the three-dimensional structural model of human rhombotin-2 (PDB entry 2xjy), with the fragment of the LID protein from PDB entry 2xjy retained**. **(a) **A surface representation of the interacting proteins. The ISL1 protein model is shown in white, while the LID protein is in red. **(b) **Secondary structure representation of the two proteins. The two LIM domains of the ISL1 protein are colored yellow and orange, with the remainder of the protein shown in purple. The LID fragment is shown in red. The crucial interaction between Tyr71 of ISL1 and Asp354 of the LID is shown by the stick representation of the two interacting side chains (indicated by the blue arrow). The green spheres correspond to the zinc atoms bound by the zinc fingers of the LIM domains. The images were generated using PyMol [90].

Thus, to help understand the structural effects of the Y71C mutation, we built a homology model for Isl1, using the rhombotin-2 protein from PDB entry 2xjy as a template. The sequence identity of the two LIM domains in the two proteins is 34% over 126 residues, giving an E-value of 9 × 10^-32^, so the model is expected to be a good approximation of the structure of Isl1. Figure 7 shows the model, with the LID from PDB entry 2xjy retained to show the interactions that one might expect between the LIM domains of Isl1 and the LIDs of the protein(s) they bind to. Of particular interest is Tyr71. The equivalent of this residue in the PDB 2xjy structure is Tyr84. This makes a side chain-side chain hydrogen bond with Asp354 in the LID of the partner protein. It turns out to be the only side chain-side chain hydrogen bonded interaction across the interface between the two proteins. In all, 12 pairs of residues interact via hydrogen bonds across this interface and all but the Tyr84-Asp354 interaction are hydrogen bonds between main chain atoms. So mutations to any of these other residues are far less likely to disrupt the binding of the two proteins. Indeed, it seems to be a feature of the LID-LIM interface that it is particularly tolerant to mutation [64]. The exception would appear to be the Tyr84-Asp354 interaction.

### Role of Isl1 in middle ear function

We propose that the Isl1 Y71C mutation leads to the predisposition of heterozygotes to develop otitis media, for several reasons. Following exome resequencing, the *Isl1 *variant was the only candidate that was confirmed by capillary sequencing. The tyrosine residue at this location is highly conserved among many species and in other mouse strains. The *Isl1 *mutation segregates with the phenotype, with all affected mice carrying the mutation in heterozygote form. No other likely pathogenic DNA changes linked to *Isl1 *on chromosome 13 were identified. Isl1 is expressed in the middle ear mucosa of wild-type mice. Finally, three-dimensional modeling of LIM domain interactions pinpoints the amino acid altered by this mutation as being particularly important in protein-protein interactions. As it was not possible to map the locus of the causative gene in dearisch using traditional backcross matings due to the low penetrance of the phenotype, exome resequencing has proved to be invaluable in identifying the likely causative mutation.

Isl1 is a transcription factor that acts as an insulin enhancing gene [65]. It contains two LIM domains and one carboxy-terminal homeodomain involved in protein-protein and protein-DNA interactions. Our modeling suggests this protein-protein interaction is likely to have been interrupted by the mutation we discovered in dearisch mutants. *Isl1 *has one isoform in mice and seven isoforms in humans and is located on chromosomes 13 and 5, respectively. Several mouse mutations affecting *Isl1 *exist, and the most widely studied is the *Isl1^tm1Tmj ^*allele [66], which consists of a neo cassette insertion into the DNA sequence encoding the second LIM domain. Mice with this mutation are homozygote lethal at embryonic day (E)11.5. Dearisch also appears to be homozygote lethal, although the age and cause for this has yet to be determined. Of four embryos so far harvested from dearisch heterozygote by heterozygote matings at E9.5, one has been genotyped as a homozygote. This pup looked immature and abnormal on external inspection (data not shown). Homozygotes of *Isl1^tm1Tmj^*exhibit malformed vasculature, including the dorsal aorta, foregut and pancreatic malformations, and exhibit no motor neuron development. Heterozygote carriers of *Isl1^tm1Tmj^*have not been reported to have any middle ear or inflammatory defects. However, Isl1 is expressed in both immature cochlear hair cells and in auditory neurons [67]. Over-expression of *Isl1 *results in protection of neurons from age-related and noise-induced hearing loss [68]. No electrophysiological studies of inner ear function in *Isl1 *mutants have been previously reported. Surprisingly, despite evidence of widespread neuronal irregularities in *Isl1 *knockout mice and the known expression of Isl1 within the inner ear, no evidence of sensorineural abnormalities were detected in the affected dearisch mice. This suggests that one copy of the wild-type *Isl1 *allele is sufficient for normal development of auditory neurons and hair cells.

Prior to this study, Isl1 expression in the middle ear had not previously been reported. However, Isl1 expression has been documented within other mucosal epithelial linings. Expression of Isl1 is strong in the ultimobranchial epithelium of the pharynx at embryonic stages [69], and was increased in specification of lung bud at E8.5 to E9.5 [70]. Isl1 expression has been found in somatostatin-expressing cells of the gastric mucosa in juvenile rats, suggesting that Isl1 may have a role in regulating somatostatin gene expression [71]. In the lungs, somatostatin is known to decrease substance-P-related mucous secretion from submucosal glands [72]. This suggests that Isl1 may affect mucous secretion from the mucosa through effects on somatostatin. We found that Isl1 is expressed in the wild-type adult middle ear mucosa. This might be expected, as middle ear mucosa is often described as being a respiratory-type mucosa. Through secretion of protective factors such as lactoferrin, lysozyme and mucus, the middle ear mucosa can reduce risk of infection [73]. Isl1 may contribute to predisposition to otitis media by affecting the constituents, amount or protective nature of middle ear mucosal secretions.

The innate immune system offers non-specific immediate defense against infection. The cytokines form part of this system, recruiting immune cells and initiating or reducing inflammation by acting as chemical mediators to specific genetic pathways. Interleukin 6 is one such cytokine. It binds the gp130 component of the type 1 cytokine receptor complex, resulting in activation of the receptor, which initiates intracellular signaling. JAK1 and STAT3 are known to be activated by this process [74]. The JAK-STAT pathway is involved in acute phase response and chronic inflammation in a variety of tissues, including the lungs and gut [75]. Isl1 has been shown to physically interact with both JAK1 and STAT3, forming a complex in both human and monkey immortal cell lines [76]. This results in the activation of STAT3, which acts as an important signal transducer and activator of transcription. JAK1 is also activated and is able to dock and recruit further signaling proteins. STAT3 has been shown to be necessary for lung and bladder epithelium to respond effectively to Gram-negative bacteria [77,78]. Without Isl1 the function of both of these genes in the prevention of infection or inflammation via innate immunity is potentially disrupted. Like *Isl1 *mutants and dearisch, *Stat3 *knockout mice suffer from embryonic lethality, while *Jak1 *knockout in mice results in perinatal mortality [79].

The importance of innate immunity in reducing otitis media is already well documented. For example, toll like receptors (Tlrs) recognize bacterial endotoxin, stimulating *TNFα *production, which in turn affects production of immunoglobulins, cytokines and mucin [46]. Mice that are genetically deficient for *Tlr4*, such as the C3H/HeJ inbred strain, develop chronic otitis media due to an inability to clear Gram-negative bacteria [32]. Between 35% and 60% of these mice were affected by otitis media at some point during their life span. Unlike affected dearisch mice, there was also evidence of bony remodeling of the round window and elements of inner ear inflammation in some C3H/HeJ mice. Knockout of *gp130 *suggests that the *Tlr4 *[31,32] pathway response to bacterial endotoxin may be modulated by the Stat3 pathway [80]. The role of Isl1 in innate immunity is yet to be fully elucidated, but wild-type Isl1 levels in heterozygote dearisch mice may be sufficiently low to reduce their ability to clear bacteria from the middle ear.

In humans, several rare point mutations in *ISL1 *have been shown to lead to maturity onset diabetes of the young [81]. An increased incidence of otitis media has not been reported in people with mutations of this gene but a general increased propensity to infection is well-recognized in diabetics. Otitis media is very common and therefore an increased prevalence of otitis media in these patients may have gone unnoticed.

## Conclusions

Dearisch mice are ENU-induced mutants that have a predisposition to otitis media associated with a tyrosine to cysteine missense mutation in *Isl1*. This results in chronic otitis media with effusion associated with non-progressive hearing impairment from 3 weeks of age. Gross and microscopic inner ear anatomies are normal and there is no evidence of sensorineural hearing impairment, suggesting that decreased levels of wild-type Isl1 do not affect inner ear function. The middle ear of affected dearisch mice shows a thickened mucosa and cellular effusion, while Isl1 is expressed in the normal middle ear mucosa. This suggests a previously unknown role for Isl1 in middle ear function. Dearisch, *Isl1^Drsh^*, represents the first point mutation in the mouse *Isl1 *gene and suggests a previously unrecognized effect of this gene. This is also the first recorded sequencing of the C3HeB/FeJ background common to many ENU mutants and highlights the use of exome resequencing in identifying mutations leading to low penetrance phenotypes.

## Materials and methods

### Origin of the dearisch mutant

The founder mouse was detected in a large-scale ENU mutagenesis program aimed at detecting new dominantly inherited phenotypes [9]. Impaired hearing was detected by screening for lack of an earflick (Preyer reflex) in response to a 20 kHz calibrated sound burst using a custom-made clickbox. Mice that did not respond were studied in more detail. Affected dearisch mice (also known as DEA2) appeared to lose their Preyer reflex from several months of age. The colony was managed by mating affected mice with unaffected littermates, and the line was maintained on the original genetic background of the mutagenized males, C3HeB/FeJ.

### Auditory brainstem responses

ABRs were measured with recovery anesthesia using three scalp electrodes [82]. Responses were recorded to broadband clicks and tone bursts at 3, 6, 12, 18, 24, 30, 36 and 42 kHz and at a wide range of intensities from 10 to 97 dB SPL in 3 dB steps. Thresholds were determined using a stack of response waveforms and identifying the lowest stimulus at which an identifiable waveform occurs. This ABR protocol was performed on 9 mice at single time points and 16 mice at 4-weekly intervals from 8 to 24 weeks. Input/output functions were then calculated using amplitude and latency of P1/N1 and P4/N4 waveform components plotted with respect to stimulus intensity (n = 13 affected and 13 unaffected mice at 3 to 15 weeks of age).

A short ABR protocol taking approximately 6 minutes per mouse, consisting of a 70 dB SPL test click, broadband clicks from 10 to 97 dB in 3 dB steps followed by a further test 70 dB SPL click and anesthetic recovery, was used to screen the entire colony (n = 85). Following this, all mice born underwent this short ABR protocol at 6 to 8 weeks of age (n = 348 in total) and the results used to plan matings. However, only mice born between 2009 and 2011 (n = 250) were analyzed for constructing frequency histograms to avoid bias due to selective retention of affected mice born before 2009. Mice with thresholds above 50 dB were defined as affected based on the population distribution of click thresholds shown in Figure 1a. This short ABR protocol was used to assess younger mice from the dearisch colony at 3, 6 and 8 weeks of age (n = 66, with 35 mice undergoing single recordings and 31 undergoing repeated ABR measurements).

An outcross was performed with an affected dearisch male and a female from the C57BL/6J inbred colony. F1 offspring (n = 168) were screened using the short ABR protocol. Affected F1 mice were mated with another C57BL/6J mouse to create a backcross. The backcross offspring from these matings (n = 77) were screened using the short ABR protocol.

A pedigree was drawn up using information from ABR tests over several generations of the dearisch colony. This has been combined with data from *Isl1 *genotyping.

### Inner ear anatomy

Inner ear clearing was performed using glycerol as described previously [83] (n = 5 affected and 5 unaffected littermate controls, aged 15 months). Round and oval window measurements were taken from images of cleared inner ears using Adobe Photoshop. Each measurement was performed four times and averaged. Scanning electron microscopy (n = 3 affected and 3 unaffected littermate controls, aged 2 months) was performed following fixation in 2.5% glutaraldehyde, a standard osmium-thiocarbohydrazide-osmium OTOTO protocol, dehydration, critical point drying and examination in a Hitachi S-4800 scanning electron microscope.

### Middle ear anatomy and immunocytochemistry

Middle ear dissections were performed on fresh tissue (n = 14 affected and 14 unaffected littermate controls, aged 9.3 to 24.0 months-mean 16.8 months, standard deviation 4.2 months) and observations were recorded on a standard tick sheet. First the tympanic membrane was inspected, the tissue covering the bulla was dissected away and the bulla inspected. The bulla was carefully removed and the tympanic membrane inspected a second time. The tympanic membrane was removed and the presence of fluid, inflamed mucosa or debris recorded. The malleus, incus and stapes were removed and photographed before removing the inner ear for clearing.

For histology, half heads of mice were fixed in 10% formalin and decalcified using EDTA for 10 days. Following alcohol dehydration the half heads were embedded in paraffin wax, sectioned to 8 μm and stained according to a standard hematoxylin/eosin protocol (n = 4 affected and 4 unaffected littermate controls, aged 6 months). Isl1 expression was inspected on sections from the same mice (n = 3 littermate controls, aged 6 months) using Isl1 antibody (AbCam: 20670, Cambridge, Cambridgeshire, UK) according to the immunohistochemistry protocol described previously [84]. Postnatal day 4 pups were also used for immunohistochemistry (n = 4 wild types), but no decalcification step was required.

### Bacteriology

Swabs from the outer and middle ear of affected and litter mate controls (n = 4 affected and 2 unaffected littermate controls, aged > 15 months) were firstly grown on nutrient broth and on L-agar plates (Oxoid Ltd, Basingstoke, UK). The bacteria were identified by plating on selective media that included CLED, MaConkey's and UTI brilliance agar (Oxoid Ltd). Oxidase testing was used as a final confirmatory step.

### Exome sequencing and analysis of the *Isl1 *mutation in the dearisch colony

One deaf dearisch mouse and one mouse of the original background C3HeB/FeJ were used for exome sequencing using a pre-market product from Agilent (Agilent SureSelect^XT ^mouse all exon kit for exome sequence capture). This uses 55,000 biotinylated cRNAs to identify the exome and surrounding intronic and intergenic information, including microRNAs. Magnetic beads are then used to pull-down the relevant DNA. Remaining DNA is washed away and the cRNA digested.

DNA (2 to 3 μg in TE) was sheared to 100 to 400 bp using a Covaris E210 (Covaris, Woburn, MA, USA). Sheared DNA was subjected to Illumina paired-end DNA library preparation according to manufacturer's recommendations (NEBNext DNA Sample Prep Set 1; New England BioLabs, Ipswich, MA, USA) and the adapter-ligated libraries were amplified for five to six cycles using Herculase II (Agilent Technologies) with PE1.0 and PE2.0 oligonucleotides (Illumina, San Diego, CA, USA). Amplified library (500 ng) was hybridized to the mouse bait library (SureSelect^XT ^Mouse All Exon Kit; Agilent Technologies, catalogue number G7500A) according to the manufacturer's recommendations. Hybridized material was captured using streptavidin-coated beads (Invitrogen, Paisley, UK) and amplified for 10 to 11 cycles using Herculase II with PE1.0 and PE2.0 oligonucleotides (Illumina). Captured libraries were sequenced on the Illumina Genome Analyzer II platform as paired-end 76-bp reads according to the manufacturer's protocol. Two lanes of sequence were generated for each mouse.

Sequence data have been deposited in the European Nucleotide Archive (accession number ERP000744).

Primers to amplify the regions containing the potential DNA changes detected by Illumina sequencing were designed using Primer 3 [85] and ordered from Sigma, (Haverhill, Suffolk, UK) (Table 6). DNA from the original sequenced mice underwent capillary sequencing to exclude false positives. The same *Isl1 *primer and capillary sequencing were used to assess mice from the rest of the dearisch colony and other C3HeB/FeJ mice. Indels and SNVs originally excluded by the final filtering step before capillary sequencing were examined along the entire length of chromosome 13 to exclude any potentially pathogenic mutation that may be linked to *Isl1*. The following wild-type mouse strains were also sequenced to establish the *Isl1 *sequence: NOR/Lt, BUB/BnJ, I/LnJ, C3HeB/FeJ, FVB/N, 129P2/OlaHsd, CBA, PL/J, 101/H, C57BL/6J, SWR/J, P/J, BALB/c, LG/J, CHMU/LeJ, MA/MyJ, SB/Le, PN/nBSwUmabJ, DBA/1J, DA/HuSn, and SM/J.

**Table 6:** Primers used for capillary sequencing of the 23 SNVs and for genotyping the *Isl1 *mutation

Gene name	Location	Forward primer	Reverse primer
1700001K19Rik	12:111907080	CTTCTTCTTCCTCTTAATTCTCTCAGG	TTTAGATCCTTATGATGGTGACTCG
1700104B16Rik	8:34841236	TGTAGCAGACTGGGCTTTGC	CTCCCTCAGTCCCTACAAGC
*Acsl3*	1:78692680	CTCTCACCTGTGTGCTCTGG	CCTGATCTGCTAATGTCTGTGG
*Bcl2l14*	6:134377474	CTTCATCCTAAACAACCAGAAGTCC	AGTGTGATTAGAGCTAGTCCTCTTCTCC
*Btnl7*	17:34670007	AGAGAGTTCCTGGCATGTGG	CAGGCTTAGGACTGGAGACG
*Catsper2*	2:121223476	CTGGATGTCTTACACTCACTACACTGC	CTATATGTACAGAGGGACCAGTCTTGG
*Col6a3*	1:92672331	CAGGCATAAAAGATGGTGTCTCTAAG	GACCAAACCAACAGCAATTGTAAAC
*Creb3l2*	6:37284584	GATGCCCTGAGCAGAGAGG	TGCAGAAAGCCAAACCTAGC
*Gm10859*	2:5833494	AATCTCAGTTGAGAGAAAACCTACG	GAGATAGCTCAGTCAGTCAGTCAGG
*Gm11149*	9:49380322	GACATTCTCTAAAAGCAGAGACATCC	ACGGACTACAGTCTAAAACATCTAAGC
*Gtf3c2*	5:31476808	CCTCAAATCCAGGCAAAGG	CTCGTTGCTGTATCTCTGTGC
H2-Oa	17:34229420	CTAACCTGGACTCTGTTTCTTTTTACC	CTACATTTCCACTGACTCTTTCAGAGC
*Ido1*	8:25703857	CCGGTAGTGGATGCTGTAGG	CTCTAAGTGACCTCCGTGAGC
*Isl1*	13:117098488	CACTGGGCACTCTAAAGTAAACG	TTCTCCGGATTTGGAGTGG
*Mdc1*	17:35984844	CATCTGCAGGACTGCCTAGC	TGGGACTTGACCTCTTCTGC
*Olfr424*	1:176066876	GGACAAAGAATAACACAGATTTTCC	GAACAAAGGAATGAAGAAGAGG
*Olfr573-ps1*	7:110091057-8	AGAGGAAGTAGTACATAGGCTCATGG	CTACTGAAAGAGTTAACTTAGTGGAGAGG
*Olfr749*	14:51356853	AGACAGAATGTTGGCTAGTATGTTAGG	CTAATTATCTAGATCGCCTTTGACTCC
*Rsf1*	7:104809403-4	GACACTAAAAGTAGAAAGCAGTCACC	GCTTTTCTAGCTTTACAATGACTGG
*Sap30bp*	11:115825338	CAACACAGGAAATGGACACG	AACCAACAGGACCCAGAGG
U1	1:172958261	TAAATACTTACCTGGCAGGAGAGATACC	TTATATTGGTGCACTAGCTTCATGC

### Three-dimensional modeling

We used the PDBsum database [86] to find all structural models containing one or more LIM domains (Pfam identifier PF00412), and then examined those having two tandem LIM domains to find any that might be in complex with a binding partner. One such was PDB entry 2xjy, solved by X-ray crystallography to 2.4 Å resolution. This is a complex of human rhombotin-2 (aka LMO2) and a 35-residue fragment of a LIM-interaction domain (LID) from human LIM domain-binding protein 1.

We used the SWISS-MODEL server [87] to build automatically a three-dimensional homology model of ISL1 using the three-dimensional structure of rhombotin-2 from PDB entry 2xjy as our template. The sequence identity of the two LIM domains in the two proteins is 34% over 126 residues, giving an E-value of 9 × 10^-32^, so the model is expected to be a good approximation of the structure of Isl1. To our model we added the LID fragment from PDB entry 2xjy (by cut-and-paste between PDB files), and noted that the Tyr84-Asp354 side chain interaction from 2xjy was retained as Tyr81-Asp354 in our model.

## Abbreviations

ABR: auditory brainstem response; bp: base pair; *Drsh*: dearisch; E: embryonic day; ENU: *N*-ethyl-*N*-nitrosourea; LID: LIM-interaction domain; LIM-HD: LIM-homeodomain; PDB: Protein Data Bank; SNP: single nucleotide polymorphism; SNV: single nucleotide variant; SPL: sound pressure level ' Tlr: toll like receptor

## Competing interests

The authors declare that they have no competing interests.

## Authors' contributions

JMH, MAL, NI, DJA and KPS designed the experiments. JMH, NI and SP carried out the ABR experiments. JMH, MAL and MG carried out the other phenotyping experiments. JMH, MAL, DJA and KPS planned and interpreted the sequence analysis. RAL carried out the modeling analysis. KPS devised the screen for new deaf mutants and directed the program. The paper was drafted by JMH, MAL, RAL and KPS and all authors contributed to the final version.

## References

[B1] Rovers MM Schilder AG Zielhuis GA Rosenfeld RM Otitis media. Lancet 2004 363 465 473 10.1016/S0140-6736(04)15495-0 14962529

[B2] Brauer M Gehring U Brunekreef B de Jongste J Gerritsen J Rovers M Wichmann HE Wijga A Heinrich J Traffic-related air pollution and otitis media. Environ Health Perspect 2006 114 1414 1418 10.1289/ehp.9089 16966098 PMC1570088

[B3] Paradise JL Rockette HE Colborn DK Bernard BS Smith CG Kurs-Lasky M Janosky JE Otitis media in 2253 Pittsburgh-area infants: prevalence and risk factors during the first two years of life. Pediatrics 1997 99 318 333 10.1542/peds.99.3.318 9041282

[B4] Teele DW Klein JO Rosner B Epidemiology of otitis media during the first seven years of life in children in greater Boston: a prospective, cohort study. J Infect Dis 1989 160 83 94 10.1093/infdis/160.1.83 2732519

[B5] Dubey SP Larawin V Complications of chronic suppurative otitis media and their management. Laryngoscope 2007 117 264 267 10.1097/01.mlg.0000249728.48588.22 17277619

[B6] Casselbrant ML Mandel EM Fall PA Rockette HE Kurs-Lasky M Bluestone CD Ferrell RE The heritability of otitis media: a twin and triplet study. JAMA 1999 282 2125 2130 10.1001/jama.282.22.2125 10591333

[B7] Daly KA Brown WM Segade F Bowden DW Keats BJ Lindgren BR Levine SC Rich SS Chronic and recurrent otitis media: a genome scan for susceptibility loci. Am J Hum Genet 2004 75 988 997 10.1086/426061 15514890 PMC1225283

[B8] Browning G Luxon L Gleeson MJ The ear, hearing and balance. Scott-Browns Otorhinolaryngology, Head and Neck Surgery 2008 3 7 Oxford: Oxford University Press 3099 3894

[B9] Hrabé de Angelis MH Flaswinkel H Fuchs H Rathkolb B Soewarto D Marschall S Heffner S Pargent W Wuensch K Jung M Reis A Richter T Alessandrini F Jakob T Fuchs E Kolb H Kremmer E Schaeble K Rollinski B Roscher A Peters C Meitinger T Strom T Steckler T Holsboer F Klopstock T Gekeler F Schindewolf C Jung T Avraham K Genome-wide, large-scale production of mutant mice by ENU mutagenesis. Nat Genet 2000 25 444 447 10.1038/78146 10932192

[B10] Hardisty RE Erven A Logan K Morse S Guionaud S Sancho-Oliver S Hunter AJ Brown SD Steel KP The deaf mouse mutant Jeff (*Jf*) is a single gene model of otitis media. J Assoc Res Otolaryngol 2003 4 130 138 10.1007/s10162-002-3015-9 12943368 PMC3202714

[B11] Parkinson N Hardisty-Hughes RE Tateossian H Tsai HT Brooker D Morse S Lalane Z MacKenzie F Fray M Glenister P Woodward AM Polley S Barbaric I Dear N Hough TA Hunter AJ Cheeseman MT Brown SD Mutation at the Evi1 locus in Junbo mice causes susceptibility to otitis media. PLoS Genet 2006 2 e149 10.1371/journal.pgen.0020149 17029558 PMC1592239

[B12] Rye MS Bhutta MF Cheeseman MT Burgner D Blackwell JM Brown SD Jamieson SE Unraveling the genetics of otitis media: from mouse to human and back again. Mamm Genome 2011 22 66 82 10.1007/s00335-010-9295-1 21107580

[B13] Hardisty-Hughes RE Tateossian H Morse SA Romero MR Middleton A Tymowska-Lalanne Z Hunter AJ Cheeseman M Brown SD A mutation in the F-box gene, *Fbxo11*, causes otitis media in the Jeff mouse. Human Mol Genet 2006 15 3273 3279 10.1093/hmg/ddl403 17035249

[B14] Tateossian H Hardisty-Hughes RE Morse S Romero MR Hilton H Dean C Brown SD Regulation of TGF-beta signalling by *Fbxo11*, the gene mutated in the Jeff otitis media mouse mutant. PathoGenet 2009 2 5 10.1186/1755-8417-2-5 PMC2714483 19580641

[B15] Rye MS Wiertsema SP Scaman ES Oommen J Sun W Francis RW Ang W Pennell CE Burgner D Richmond P Vijayasekaran S Coates HL Brown SD Blackwell JM Jamieson SE FBXO11, a regulator of the TGFbeta pathway, is associated with severe otitis media in Western Australian children. Genes Immun 2011 12 352 359 10.1038/gene.2011.2 21293382

[B16] Pau H Fuchs H Hrabé de Angelis M Steel KP Hush puppy: a new mouse mutant with pinna, ossicle, and inner ear defects. Laryngoscope 2005 115 116 124 10.1097/01.mlg.0000150693.31130.a0 15630379

[B17] Calvert JA Dedos SG Hawker K Fleming M Lewis MA Steel KP A missense mutation in *Fgfr1 *causes ear and skull defects in hush puppy mice. Mamm Genome 2011 22 290 305 10.1007/s00335-011-9324-8 21479780 PMC3099004

[B18] Yang A Walker N Bronson R Kaghad M Oosterwegel M Bonnin J Vagner C Bonnet H Dikkes P Sharpe A McKeon F Caput D p73-deficient mice have neurological, pheromonal and inflammatory defects but lack spontaneous tumours. Nature 2000 404 99 103 10.1038/35003607 10716451

[B19] Schmidt-Ullrich R Aebischer T Hulsken J Birchmeier W Klemm U Scheidereit C Requirement of NF-kappaB/Rel for the development of hair follicles and other epidermal appendices. Development 2001 128 3843 3853 11585809 10.1242/dev.128.19.3843

[B20] Humbert PO Rogers C Ganiatsas S Landsberg RL Trimarchi JM Dandapani S Brugnara C Erdman S Schrenzel M Bronson RT Lees JA E2F4 is essential for normal erythrocyte maturation and neonatal viability. Mol Cell 2000 6 281 291 10.1016/S1097-2765(00)00029-0 10983976

[B21] Depreux FF Darrow K Conner DA Eavey RD Liberman MC Seidman CE Seidman JG Eya4-deficient mice are a model for heritable otitis media. J Clin Invest 2008 118 651 658 18219393 10.1172/JCI32899 PMC2213371

[B22] Giovannini M Robanus-Maandag E van der Valk M Niwa-Kawakita M Abramowski V Goutebroze L Woodruff JM Berns A Thomas G Conditional biallelic Nf2 mutation in the mouse promotes manifestations of human neurofibromatosis type 2. Genes Dev 2000 14 1617 1630 10887156 PMC316733

[B23] Eriksson PO Li J Ny T Hellstrom S Spontaneous development of otitis media in plasminogen-deficient mice. Int J Med Microbiol 2006 296 501 509 10.1016/j.ijmm.2006.04.002 16956791

[B24] Liao J Kochilas L Nowotschin S Arnold JS Aggarwal VS Epstein JA Brown MC Adams J Morrow BE Full spectrum of malformations in velo-cardio-facial syndrome/DiGeorge syndrome mouse models by altering Tbx1 dosage. Hum Mol Genet 2004 13 1577 1585 10.1093/hmg/ddh176 15190012

[B25] Noben-Trauth K Latoche JR Ectopic mineralization in the middle ear and chronic otitis media with effusion caused by RPL38 deficiency in the Tail-short (*Ts*) mouse. J Biol Chem 2011 286 3079 3093 10.1074/jbc.M110.184598 21062742 PMC3024801

[B26] Wang L Bresee CS Jiang H He W Ren T Schweitzer R Brigande JV Scleraxis is required for differentiation of the stapedius and tensor tympani tendons of the middle ear. J Assoc Res Otolaryngol 2011 12 407 421 10.1007/s10162-011-0264-5 21399989 PMC3123444

[B27] Warren M Wang W Spiden S Chen-Murchie D Tannahill D Steel KP Bradley A A *Sall4 *mutant mouse model useful for studying the role of *Sall4 *in early embryonic development and organogenesis. Genesis 2007 45 51 58 10.1002/dvg.20264 17216607 PMC2593393

[B28] Mao M Thedens DR Chang B Harris BS Zheng QY Johnson KR Donahue LR Anderson MG The podosomal-adaptor protein SH3PXD2B is essential for normal postnatal development. Mamm Genome 2009 20 462 475 10.1007/s00335-009-9210-9 19669234 PMC2759419

[B29] Megerian CA Semaan MT Aftab S Kisley LB Zheng QY Pawlowski KS Wright CG Alagramam KN A mouse model with postnatal endolymphatic hydrops and hearing loss. Hear Res 2008 237 90 105 10.1016/j.heares.2008.01.002 18289812 PMC2858221

[B30] Leichtle A Hernandez M Pak K Yamasaki K Cheng CF Webster NJ Ryan AF Wasserman SI TLR4-mediated induction of TLR2 signaling is critical in the pathogenesis and resolution of otitis media. Innate Immun 2009 15 205 215 10.1177/1753425909103170 19586996 PMC3565845

[B31] Hirano T Kodama S Fujita K Maeda K Suzuki M Role of Toll-like receptor 4 in innate immune responses in a mouse model of acute otitis media. FEMS Immunol Med Microbiol 2007 49 75 83 10.1111/j.1574-695X.2006.00186.x 17266713

[B32] MacArthur CJ Hefeneider SH Kempton JB Trune DR C3H/HeJ mouse model for spontaneous chronic otitis media. Laryngoscope 2006 116 1071 1079 10.1097/01.mlg.0000224527.41288.c4 16826039

[B33] Hernandez M Leichtle A Pak K Ebmeyer J Euteneuer S Obonyo M Guiney DG Webster NJ Broide DH Ryan AF Wasserman SI Myeloid differentiation primary response gene 88 is required for the resolution of otitis media. J Infect Dis 2008 198 1862 1869 10.1086/593213 18986247 PMC3584630

[B34] Leichtle A Hernandez M Pak K Webster NJ Wasserman SI Ryan AF The toll-Like receptor adaptor TRIF contributes to otitis media pathogenesis and recovery. BMC Immunol 2009 10 45 10.1186/1471-2172-10-45 19656404 PMC2736931

[B35] Rivkin AZ Palacios SD Pak K Bennett T Ryan AF The role of Fas-mediated apoptosis in otitis media: observations in the *lpr/lpr *mouse. Hear Res 2005 207 110 116 10.1016/j.heares.2005.04.010 15978756

[B36] Vogler C Levy B Galvin N Sands MS Birkenmeier EH Sly WS Barker J A novel model of murine mucopolysaccharidosis type VII due to an intracisternal a particle element transposition into the beta-glucuronidase gene: clinical and pathologic findings. Pediatr Res 2001 49 342 348 10.1203/00006450-200103000-00007 11228259

[B37] Schachern PA Cureoglu S Tsuprun V Paparella MM Whitley CB Age-related functional and histopathological changes of the ear in the MPS I mouse. Int J Pediatr Otorhinolaryngol 2007 71 197 203 10.1016/j.ijporl.2006.09.016 17101178 PMC1940035

[B38] Heldermon CD Hennig AK Ohlemiller KK Ogilvie JM Herzog ED Breidenbach A Vogler C Wozniak DF Sands MS Development of sensory, motor and behavioral deficits in the murine model of Sanfilippo syndrome type B. PLoS One 2007 2 e772 10.1371/journal.pone.0000772 17712420 PMC1945015

[B39] Voronina VA Takemaru K Treuting P Love D Grubb BR Hajjar AM Adams A Li FQ Moon RT Inactivation of Chibby affects function of motile airway cilia. J Cell Biol 2009 185 225 233 10.1083/jcb.200809144 19364920 PMC2700371

[B40] Ibanez-Tallon I Gorokhova S Heintz N Loss of function of axonemal dynein Mdnah5 causes primary ciliary dyskinesia and hydrocephalus. Hum Mol Genet 2002 11 715 721 10.1093/hmg/11.6.715 11912187

[B41] Han F Yu H Zhang J Tian C Schmidt C Nava C Davisson MT Zheng QY Otitis media in a mouse model for Down syndrome. Int J Exp Pathol 2009 90 480 488 10.1111/j.1365-2613.2009.00677.x 19765102 PMC2768146

[B42] Alpay HC Etem EO Kaygusuz I Yuce H Karlidag T Keles E Orhan I Yalcin S Evaluation of the polymorphism in the Toll-like receptor 4 (*TLR4*) genes of tympanosclerosis patients. Auris Nasus Larynx 2010 37 29 32 10.1016/j.anl.2009.03.001 19398177

[B43] Ubell ML Khampang P Kerschner JE Mucin gene polymorphisms in otitis media patients. Laryngoscope 2010 120 132 138 10.1002/lary.21596 19718741 PMC2919485

[B44] Alper CM Winther B Hendley JO Doyle WJ Cytokine polymorphisms predict the frequency of otitis media as a complication of rhinovirus and RSV infections in children. Eur Arch Otorhinolaryngol 2009 266 199 205 10.1007/s00405-008-0729-2 18560870 PMC7087847

[B45] Patel JA Nair S Revai K Grady J Saeed K Matalon R Block S Chonmaitree T Association of proinflammatory cytokine gene polymorphisms with susceptibility to otitis media. Pediatrics 2006 118 2273 2279 10.1542/peds.2006-0764 17142509

[B46] Emonts M Veenhoven RH Wiertsema SP Houwing-Duistermaat JJ Walraven V de Groot R Hermans PW Sanders EA Genetic polymorphisms in immunoresponse genes *TNFA, IL6, IL10*, and *TLR4 *are associated with recurrent acute otitis media. Pediatrics 2007 120 814 823 10.1542/peds.2007-0524 17908769

[B47] Nuytinck L De Meester E Van Thielen M Govaerts P Role of mannose-binding lectin (*MBL2*) genotyping in predicting the risk of recurrent otitis media (rOM). Adv Exp Med Biol 2006 586 281 290 10.1007/0-387-34134-X_19 16893079

[B48] Pettigrew MM Gent JF Zhu Y Triche EW Belanger KD Holford TR Bracken MB Leaderer BP Association of surfactant protein A polymorphisms with otitis media in infants at risk for asthma. BMC Med Genet 2006 7 68 10.1186/1471-2350-7-68 16884531 PMC1557482

[B49] Wiertsema SP Khoo SK Baynam G Veenhoven RH Laing IA Zielhuis GA Rijkers GT Goldblatt J Lesouef PN Sanders EA Association of CD14 promoter polymorphism with otitis media and pneumococcal vaccine responses. Clin Vaccine Immunol 2006 13 892 897 10.1128/CVI.00100-06 16893989 PMC1539116

[B50] Kalm O Johnson U Prellner K Ninn K HLA frequency in patients with recurrent acute otitis media. Arch Otolaryngol Head Neck Surg 1991 117 1296 1299 1747237 10.1001/archotol.1991.01870230112019

[B51] Prellner K Hallberg T Kalm O Mansson B Recurrent otitis media: genetic immunoglobulin markers in children and their parents. Int J Pediatr Otorhinolaryngol 1985 9 219 225 10.1016/S0165-5876(85)80037-9 3932246

[B52] Biesecker LG Exome sequencing makes medical genomics a reality. Nat Genet 2010 42 13 14 10.1038/ng0110-13 20037612

[B53] Ng SB Nickerson DA Bamshad MJ Shendure J Massively parallel sequencing and rare disease. Hum Mol Genet 2010 19 R119 124 10.1093/hmg/ddq390 20846941 PMC2953741

[B54] Agilent SureSelect Target Enrichment. http://www.agilent.com/genomics/sureselect

[B55] Li H Durbin R Fast and accurate short read alignment with Burrows-Wheeler transform. Bioinformatics 2009 25 1754 1760 10.1093/bioinformatics/btp324 19451168 PMC2705234

[B56] Picard. http://picard.sourceforge.net

[B57] Li H Handsaker B Wysoker A Fennell T Ruan J Homer N Marth G Abecasis G Durbin R The Sequence Alignment/Map format and SAMtools. Bioinformatics 2009 25 2078 2079 10.1093/bioinformatics/btp352 19505943 PMC2723002

[B58] dbSNP. http://www.ncbi.nlm.nih.gov/SNP/

[B59] Sherry ST Ward MH Kholodov M Baker J Phan L Smigielski EM Sirotkin K dbSNP: the NCBI database of genetic variation. Nucleic Acids Res 2001 29 308 311 10.1093/nar/29.1.308 11125122 PMC29783

[B60] The Mouse Genome Project. http://www.sanger.ac.uk/resources/mouse/genomes/

[B61] Finn RD Tate J Mistry J Coggill PC Sammut SJ Hotz HR Ceric G Forslund K Eddy SR Sonnhammer EL Bateman A The Pfam protein families database. Nucleic Acids Res 2010 38 D211 222 10.1093/nar/gkp985 19920124 PMC2808889

[B62] Gill GN Decoding the LIM development code. Trans Am Clin Climatol Assoc 2003 114 179 189 12813919 PMC2194522

[B63] Berman H Henrick K Nakamura H Markley JL The worldwide Protein Data Bank (wwPDB): ensuring a single, uniform archive of PDB data. Nucleic Acids Res 2007 35 D301 303 10.1093/nar/gkl971 17142228 PMC1669775

[B64] Deane JE Ryan DP Sunde M Maher MJ Guss JM Visvader JE Matthews JM Tandem LIM domains provide synergistic binding in the LMO4:Ldb1 complex. EMBO J 2004 23 3589 3598 10.1038/sj.emboj.7600376 15343268 PMC517615

[B65] Zhang H Wang WP Guo T Yang JC Chen P Ma KT Guan YF Zhou CY The LIM-homeodomain protein ISL1 activates insulin gene promoter directly through synergy with BETA2. Jof Mol Biol 2009 392 566 577 10.1016/j.jmb.2009.07.036 19619559

[B66] Pfaff SL Mendelsohn M Stewart CL Edlund T Jessell TM Requirement for LIM homeobox gene *Isl1 *in motor neuron generation reveals a motor neuron-dependent step in interneuron differentiation. Cell 1996 84 309 320 10.1016/S0092-8674(00)80985-X 8565076

[B67] Radde-Gallwitz K Pan L Gan L Lin X Segil N Chen P Expression of Islet1 marks the sensory and neuronal lineages in the mammalian inner ear. J Comp Neurol 2004 477 412 421 10.1002/cne.20257 15329890 PMC4158841

[B68] Huang M Kantardzhieva A Liberman MC Chen Z Hair-cell specific *Isl1 *transgenic mice are protected from age-related (ARHL) and noice-induced hearing loss (NIHL). Abstracts of the Thirty-fourth Annual Midwinter Research Meeting of the Association for Research in Otolaryngology: February 19-23 2011 Baltimore, MD, USA. Mt Royal, NJ: Association for Research in Otolaryngology http://www.aro.org/mwm/documents/2011_Abstract_Book.pdf 2011:abstract 139

[B69] Westerlund J Andersson L Carlsson T Zoppoli P Fagman H Nilsson M Expression of Islet1 in thyroid development related to budding, migration, and fusion of primordia. Dev Dyn 2008 237 3820 3829 10.1002/dvdy.21772 18985716

[B70] Millien G Beane J Lenburg M Tsao PN Lu J Spira A Ramirez MI Characterization of the mid-foregut transcriptome identifies genes regulated during lung bud induction. Gene Expr Patterns 2008 8 124 139 10.1016/j.modgep.2007.09.003 18023262 PMC2440337

[B71] Larsson LI Tingstedt JE Madsen OD Serup P Hougaard DM The LIM-homeodomain protein Isl-1 segregates with somatostatin but not with gastrin expression during differentiation of somatostatin/gastrin precursor cells. Endocrine 1995 3 519 524 10.1007/BF02738827 21153208

[B72] Wagner U Fehmann HC Bredenbroker D Yu F Barth PJ von Wichert P Galanin and somatostatin inhibition of substance P-induced airway mucus secretion in the rat. Neuropeptides 1995 28 59 64 10.1016/0143-4179(95)90075-6 7538203

[B73] Lim DJ Chun YM Lee HY Moon SK Chang KH Li JD Andalibi A Cell biology of tubotympanum in relation to pathogenesis of otitis media-a review. Vaccine 2000 19 Suppl 1 S17 25 11163458 10.1016/s0264-410x(00)00273-5

[B74] Hirano T Ishihara K Hibi M Roles of STAT3 in mediating the cell growth, differentiation and survival signals relayed through the IL-6 family of cytokine receptors. Oncogene 2000 19 2548 2556 10.1038/sj.onc.1203551 10851053

[B75] Neurath MF Finotto S IL-6 signaling in autoimmunity, chronic inflammation and inflammation-associated cancer. Cytokine Growth Factor Rev 2011 22 83 89 10.1016/j.cytogfr.2011.02.003 21377916

[B76] Hao A Novotny-Diermayr V Bian W Lin B Lim CP Jing N Cao X The LIM/homeodomain protein Islet1 recruits Janus tyrosine kinases and signal transducer and activator of transcription 3 and stimulates their activities. Mol Biol Cell 2005 16 1569 1583 10.1091/mbc.E04-08-0664 15659653 PMC1073642

[B77] Quinton LJ Jones MR Robson BE Simms BT Whitsett JA Mizgerd JP Alveolar epithelial STAT3, IL-6 family cytokines, and host defense during *Escherichia coli *pneumonia. Am J Resp Cell Mol Biol 2008 38 699 706 10.1165/rcmb.2007-0365OC PMC2396249 18192501

[B78] Wood MW Breitschwerdt EB Gookin JL Autocrine effects of interleukin-6 mediate acute-phase proinflammatory and tissue-reparative transcriptional responses of canine bladder mucosa. Infect Immun 2011 79 708 715 10.1128/IAI.01102-10 21115724 PMC3028824

[B79] Schindler CW Series introduction. JAK-STAT signaling in human disease. J Clin Invest 2002 109 1133 1137 10.1172/JCI15644 11994400 PMC150971

[B80] Greenhill CJ Rose-John S Lissilaa R Ferlin W Ernst M Hertzog PJ Mansell A Jenkins BJ IL-6 trans-signaling modulates TLR4-dependent inflammatory responses via STAT3. J Immunol 2011 186 1199 1208 10.4049/jimmunol.1002971 21148800

[B81] Shimomura H Sanke T Hanabusa T Tsunoda K Furuta H Nanjo K Nonsense mutation of islet-1 gene (Q310X) found in a type 2 diabetic patient with a strong family history. Diabetes 2000 49 1597 1600 10.2337/diabetes.49.9.1597 10969846

[B82] Ingham N Pearson S Steel KP Using the auditory brainstem responses to determine sensitivity of hearing in mutant mice. Curr Protoc Mouse Biol 2011 in press 10.1002/9780470942390.mo110059 26069055

[B83] Steel KP Smith RJ Normal hearing in Splotch (*Sp/+*), the mouse homologue of Waardenburg syndrome type 1. Nat Genet 1992 2 75 79 10.1038/ng0992-75 1303254

[B84] Lewis MA Quint E Glazier AM Fuchs H De Angelis MH Langford C van Dongen S Abreu-Goodger C Piipari M Redshaw N Dalmay T Moreno-Pelayo MA Enright AJ Steel KP An ENU-induced mutation of miR-96 associated with progressive hearing loss in mice. Nat Genet 2009 41 614 618 10.1038/ng.369 19363478 PMC2705913

[B85] Rozen S Skaletsky H Primer3 on the WWW for general users and for biologist programmers. Methods Mol Biol 2000 132 365 386 10547847 10.1385/1-59259-192-2:365

[B86] Laskowski RA PDBsum new things. Nucleic Acids Res 2009 37 D355 359 10.1093/nar/gkn860 18996896 PMC2686501

[B87] Schwede T Kopp J Guex N Peitsch MC SWISS-MODEL: an automated protein homology-modeling server. Nucleic Acids Res 2003 31 3381 3385 10.1093/nar/gkg520 12824332 PMC168927

[B88] Ensembl Genome Browser. http://www.ensembl.org

[B89] The ConSurf Server. http://consurf.tau.ac.il/

[B90] PyMOL. http://www.pymol.org/

